# Limitations and consequences of public health models centred on hospitals and lacking connections with territorial and home-based social and health services

**DOI:** 10.1186/s12245-024-00641-1

**Published:** 2024-05-23

**Authors:** Lavinia Gentile, Martina Scaramella, Giuseppe Liotta, Andrea Magrini, Maria Franca Mulas, Giuseppe Quintavalle, Leonardo Palombi

**Affiliations:** 1 School of Specialization in Hygiene and Preventive Medicine, University of Rome “Tor Vergata”, Rome, Italy; 2 Department of Biomedicine and Prevention, University of Rome “Tor Vergata”, Rome, Italy; 3 General Direction, University Hospital “Tor Vergata”, Rome, Italy; 4 Our Lady of Good Councel University, Tirana, Albania

**Keywords:** Frequent users, Emergency Department, Emergency Room, Public Health Model, Triage, Social frailty

## Abstract

**Background:**

Delayed discharge from hospital to home or other care institutions is a significant problem and has been investigated in the international scientific literature for many years.

Behind this condition is a health care system based on a hospital-centered concept characterized by a lack of territorial health and social welfare services.

This phenomenon causes two different problems: an excessive length of hospital stay, resulting in slow turnover of bed utilization; and overcrowding in emergency rooms (ERs).

The phenomenon of frequent users assumes particular importance in this context. These patients repeatedly visit the emergency department (ED) in the same year because care needs are not met by primary care services.

The authors in this study tried to describe the Frequent users (FUs) population and the variables associated with this condition.

**Materials and methods:**

A retrospective "single-arm" descriptive study was conducted by analysing all accesses made to the ED of Policlinico Tor Vergata (PTV) from January 1, 2022, to December 31, 2022.

FUs were defined as patients who had 4 or more accesses to PTV ER during the year.

**Results:**

A total of 37,800 accesses occurred during the study period. A total of 31,691 users accessed the PS, with a mean age of 55.8 ± 22.2 years.

There were 359 FU patients (approximately 1%) who had a total of 1984 accesses, corresponding to 5.2% of the total accesses.

The triage codes for the FU patients were red, 2%; orange, 21%; blue, 45%; green, 26%; white, 5%; and not performed, 1%.

Considering the 1984 FU accesses, the most frequently attributed "main problems" in the ED were "other symptoms or disorders" (54%), "psychomotor agitation" (12%), "trauma or burn" (8%), "abdominal pain" (6%), "chest pain" (4%), "dyspnea" (4%) and "urological symptoms or disorders" (4%). Multivariate analysis revealed that the main determinants of FUs were psychomotor agitation (HR = 7,23; CL95%:6,194–8,443), urological disorders (HR = 2,16; CL95%:1,68–2,76) and poor socioeconomic status (HR = 2,40; CL95%:2,213–2,663).

**Conclusions:**

The FUs phenomenon expresses an area of health and social distress where poverty and lack of territorial services oblige people to refer to the ED. Primary care interventions integrated with social support are crucial for managing access to the ED.

## Introduction

The overuse of resources in medical care is a problem that affects most developed and resource-limited countries. In the United States, overuse of medical services is estimated to cost $780 billion, much of which is used in hospitals [1]. The European Union lists among its priorities "Investing in health infrastructure that fosters a transformational change in the health system, in particular reinforcing the shift from a hospital-centred model to community-based care and integrated services" [2]. However, the National Health Service (NHS) in Italy is still heavily focused on the activities of hospitals and residential care more generally. The percentage share of hospital expenditure in total health expenditure is, for Italy, among the highest in the European Union: "In 2016, current hospital expenditure represented approximately 38 percent of total current health expenditure, ranging from 29 and 32 percent in Germany and Latvia, respectively, to 46 percent and 47 percent in Italy and Estonia" [3]. In 2020, hospital expenditures in Germany remained less than 30%, and hospital expenditures in Italy remained above 40% of total health expenditures [4].

This article aims to investigate the anomalies associated with the lack of territorial services. A specific phenomenon is that of frequent users (FUs), i.e., those patients who repeatedly come to the ED in the same year for various reasons and who manifest a lack of stabilization of clinical pictures or who come to the ED because of care needs that are not met by primary care services.

Many studies have attempted to classify FU patients according to many characteristics. A recurrent definition of this condition is patients who make 4 or more visits to the ED within a 12-month period [5–7]. The literature indicates that the reasons for ED overuse could be 24-h free access to care and the availability of high-tech equipment combined with the lack of effective primary care, especially for populations with lower socioeconomic status (SES) [8]. Moreover, FU patients exhibit a bimodal age distribution, with peaks in the 25 to 44 years and > 65 years age groups [9]. The older adult population seems to be highly involved in the FU phenomenon: according to more recent publications, FU patients are often older than 65 years of age, not highly educated, from low-income families, suffer from one or more chronic conditions, have at least one hospitalization within a year, and often suffer from psychiatric conditions [10]. Further studies suggest that although the cause of frequent ED use is multifactorial and thus FU patients are on average affected by underlying chronic conditions, there is a significant association with psychosocial morbidity, with perceived lower levels of social support than non-FU patients [11].

In recent years, interest has increased in the multidimensional concept of frailty, not only physically but also psychologically and socially, in line with person-centered care [12]. Empirical results suggest that despite the relevance of demographic factors, other conditions of vulnerability (poverty, alcohol and drug abuse, chronic conditions, and psychological distress) are responsible for the disproportionate absorption of resources compared to the total amount of ED costs (these costs account for approximately 10% of total patients but contribute more than 19% of the total annual ED costs) [9].

The purpose of our study was to describe and identify the sociodemographic, clinical, and health variables independently associated with FU status during 2022 and to assess the impact of FUs on EDs in the context of an area of Rome characterized by poverty, youth distress, unemployment, and scarcity of territorial services [13].

## Materials and methods

### Population

This was a retrospective "single arm” descriptive study conducted by analysing all accesses made to the Emergency Department of the Polyclinic “Tor Vergata” (PTV) from January 1 to December 31, 2022.

The Ethics Committee approved this study.

PTV is a first-level ED and a point of reference (Hub) for specific pathologies (pathological conditions such as trauma and neurotrauma, cardiological emergencies and stroke) without obstetric or pediatric units.

The data were extracted from the S.I.E.S. (Sistema Informativo Emergenze Sanitarie–Health Emergency Information System) that records all the accesses to the ER, using the variable coding system as prescribed by the relevant regulations [14] and the National and Regional Guidelines for International Classification of Diseases (ICD-9-CM) coding [15].

All the accesses to ER during 2022 have been taken into consideration. Frequent Users were defined as patients with 4 or more accesses to the PTV ED during the calendar year. The variables considered included sex, age, triage code, municipality of affiliation, discharge diagnosis, and outcome of the visit.

Triage codes were assigned according to 5 triage classes and assessed by qualified nurses based on current Italian guidelines [16], with values ranging from 1 (red) to 5 (white), where 1 indicates the highest level of severity.

### Variables considered and statistics

Data analysis was performed with Microsoft Excel v.2023 and SPSS (Illinois, Chicago, vers.26).

Descriptive statistics (mean, median, standard deviation [SD], range, frequency and percentage) were calculated for all the variables.

Univariate and multivariate logistic regression analyses were used to explore potential associations between the variables considered and the FU condition.

The level of statistical significance was set at *p* < 0.01.

## Results

A total of 37,800 accesses occurred during the study period. A total of 31,691 users accessed the ED, with a mean age of 55.8 ± 22.2 years.

There were 359 FUs (approximately 1%) and a total of 1984 accessions, corresponding to 5.2% of the total accessions.

In the FU sample, the mean age was 55.56 ± 18.48 years (65% > 65 years), and 67% were males, 84% were Italians, and 76% were residents of Municipality VI.

The general characteristics of the FUs are summarized in Table 1.

**Table 1:** Characteristics of frequent user patients

**Gender No. (%)**		**Citizenship No. (%)**		**Age No. (%)**	
males	239 (67%)	italian	303 (84%)	≥ 65 years	125 (35%)
females	120 (33%)	no italian	63 (16%)	< 65 years	234 (65%)
**Provenance N (%)**					
Municipality VI			272 (76%)		
Municipality VII			51 (14%)		
Municipality V			25 (7%)		
Municipality IV			11 (3%)		

The frequency of triage codes in the population who had at least 1 access to the PTV ED was 5% (red), 19% (orange), 41% (blue), 30% (green), 4% (white), and not performed 1%.

The triage codes for the FU patients were red, 2%; orange, 21%; blue, 45%; green, 26%; white, 5%; and not performed, 1%.

Comparing the general population with the FU sample, it can be seen that blue is the most frequently assigned triage code in both cases, followed by green and orange.

**Fig. 1:**
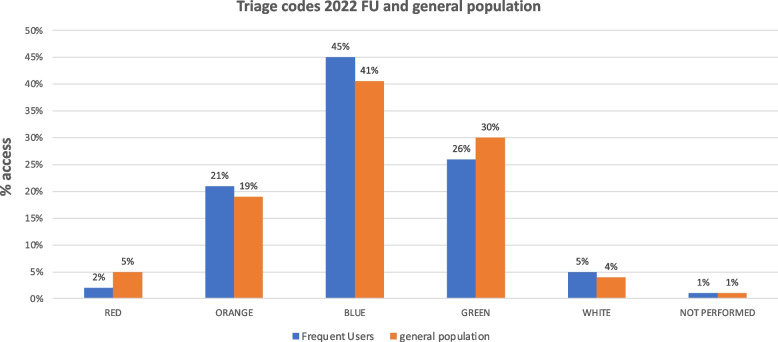
Comparison of the distribution of triage codes attributed to the general population and triage codes attributed to frequent users 2022

Overall (Fig. 1), fUs consisted of 24% red and orange codes (23% controls), while there was a greater representation of blue codes (45 vs 40%) and a lower representation of green codes (26 vs 30%).

**Fig. 2:**
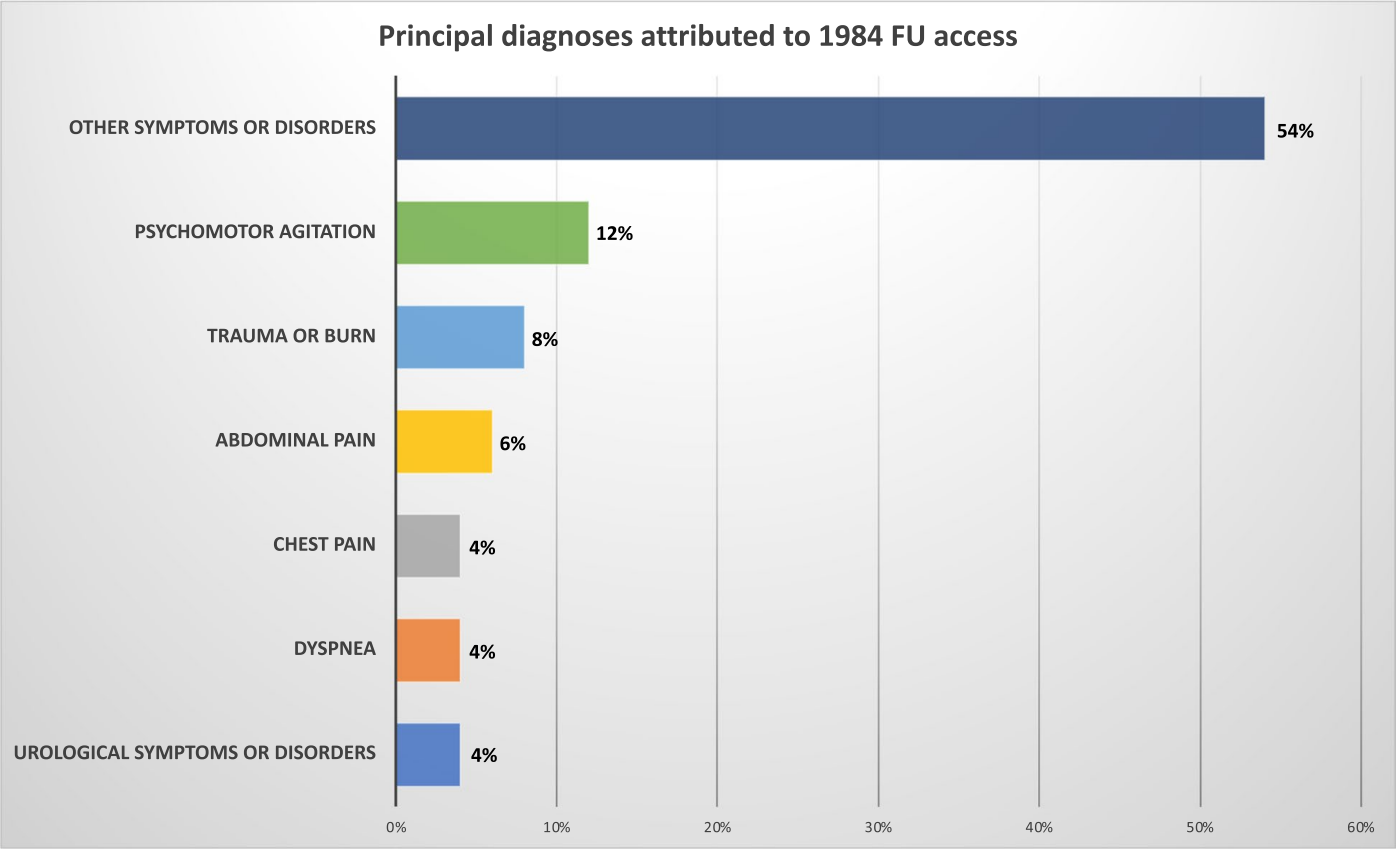
The "main problem” was most often attributed to 1984 FU accesses in EDs

The principal diagnoses attributed to FU access are depicted in Fig. 2.

As shown in Fig. 2, considering the 1984 FU visits, the most frequently attributed "main problems” in the ED were "other symptoms or disorders” (54%), "psychomotor agitation” (12%), “trauma or burn” (8%), "abdominal pain” (6%), “chest pain” (4%), “dyspnea” (4%), and “urological symptoms or disorders” (4%).

**Fig. 3:**
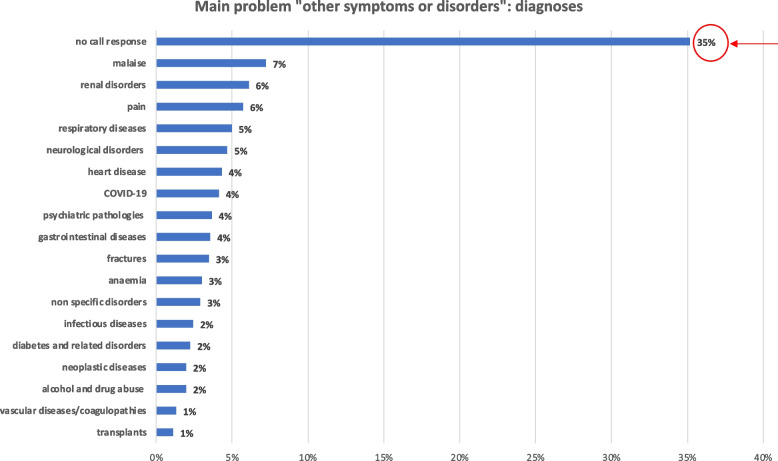
Diagnoses contained in the main problem "other symptoms or disorders”

Considering the diagnoses contained within “other symptoms or disorders” (Fig. 3), it was found that in a significant 35% of the patients, there was no call response from patients who were already triaged; therefore, the clinical problem could not be defined. Among these patients, 75% were male and 86% were Italian citizens; the triage codes assigned to these patients were white (18%), green (39%), blue (31%), orange (7%) or no red. Thus, it is inferred that the share of ED dropouts mostly involves patients of working age, especially during daytime hours, and is likely related to excessively long waits (average 1.43 ± 2.8 days).

The main problem at admission, "psychomotor agitation", was second in order of frequency, with 241 admissions. At triage entry, code green was assigned in 13% of patients, blue in 45%, orange in 38% and red in 3%. An analysis of the outcomes revealed that more than 70% of the patients did not respond to calls or left spontaneously, and 5% refused admission. These results showed that despite having been classified as having more serious cases than the average number of accesses generated by FUs, more than 75% of the accesses carried out for this cause were not followed by a continuum of diagnostic-therapeutic processes, thus resulting in nonfinalized use of ED resources.

In addition to being the main problem at admission, admissions for neurological disorders (3.9%), psychiatric pathologies (2%), and alcohol and drug abuse (1.1%) were identified as “psychomotor agitation” (12%), revealing an important area of criticality and frailty. These data reveal the need for ongoing care support focusing on the sphere of mental health and biopsychosocial well-being.

In addition, considering the prevalence of “neoplastic disease” (1.1%) and “diabetes and related disorder” (1.2%), hospitals could use diagnostic and therapeutic care pathways (PDTA) and, in particular, multidisciplinary teams for simultaneous care provision to oncological, chronic patients at risk of relapse or multiple pathological patients to reduce their frequent use of the ED.

### FU admissions

A total of 359 frequent users were diagnosed during 2022, generating 375 hospitalizations. The ED triage codes assigned to patients with hospitalization outcomes were red (8%), orange (32%), blue (45%) and green (15%).

Analysis of the SDO flow of these hospitalizations revealed that no. 191 FUs were admitted 1 to 5 times during the year at the PTV, 72% of whom were male and 28% of whom were female, generating a total of approximately 3604 inpatient days (Table 2).

**Table 2:** Association of FU access with hospitalization outcome and triage code

Gender	N° patients	Age (average/years)	Total hospital days (2022)
**male**	138 (72%)	62 ± 17	2393
**female**	53 (28%)	61 ± 19	1211

In addition, at a follow-up in March 2023, 10% of the Frequent Users identified in 2022 were found to be deceased, while 7.5% were not assigned to any GP.

Tables 3, 4 and 5 show the results of the logistic regression performed for each of the 3 categories of variables identified.

**Table 3:** Binary logistic regression–Sociodemographic variables

Variables^a^	Univariate OR (95%CI)	p	Multivariate OR (95%CI)	p
**Citizenship**		NS	1,3 (1,105–1,430)	< 0,001
**Municipality VI**	2,43 (2,212 – 2,659)	< 0,001	2,40 (2,213–2,663)	< 0,001
**Age**	0.75 (0,681 – 0,830)	< 0,001	0,80 (0.709—0.869))	< 0,001
**Gender**	0.603 (0.548 – 0.665)	< 0,001	0.609 (0.553 – 0.671)	< 0,001

^a^citizenship: 0 = foreign, 1 = Italian

City Hall VI: 0 = other city hall, 1 = City Hall VI

Age: 0 =  < 65 years old; 1 =  > 65 years old

Gender: 0 = male, 1 = female

**Table 4:** Binary logistic regression–Clinical variables

Variables	Univariate OR (95%CI)	p	Multivariate OR (95%CI)	p
**Urological disorders**	2,16 (1,686–2,762)	< 0,001	1,85 (1,445–2,376)	< 0,001
**Psychomotor agitation**	7,23 (6,194–8,443)	< 0,001	5,67 (4,847–6,636)	< 0,001
**Abdominal pain**	0,82 (0,682–0,992)	0,04	0,730 (0,604– 0,882)	0,001
**Trauma**	0,0268 (0.228– 0.314)	< 0,001	0,288 (0,245- 0,339)	< 0,001
**Neurological disorders**	0,355 (0,242- 0,521)	< 0,001	0,319 (0,217- 0,468)	< 0,001

**Table 5:** Binary logistic regression– Care outcomes

Variables	Univariate OR (95%CI)	p	Multivariate OR (95%CI)	p
**Home discharge**	0,861(-0,775- 0,958)	0,006		NS
**Hospitalization**	0,787 (0,701- 0.883)	< 0,001		NS
**Transfer**		NS		NS
**Patient refuses hospitalization**		NS		NS
**Not responding to call**	1,94 (1,750–2,145)	< 0,001	1,96 (1,759–2.175)	< 0,001
**Patient spontaneously leaves**	3,94 (3,263–4,746)	< 0,001	4,36 (3,596–5,273)	< 0,001
**Discharge to outpatient facilities**	0,538 (0,464–0,622)	< 0,001	-0,684 (0,588–0,796)	< 0,001

### Determinants of FU status

#### Sociodemographic variables

Belonging to the Municipality VI was found to be significantly associated with FU status in both univariate [OR = 2.43 (2.212–2.659)] and multivariate [OR = 2.42 (2.213–2.663)] analyses.

In contrast, age > 65 years and female sex reduced the probability of FU, according to univariate analysis [OR = 0.75 (0.681–0.830) and OR = 0.603 (0.548- 0.665), respectively], and multivariate analysis [OR = 0.80 (0.709 – 0.869) and OR = 0.609 (0.553—0.671), respectively].

Italian citizenship status, which was not significant according to the univariate analysis, was associated with FU status according to the multivariate analysis [OR = 1.3 (1.105–1.430)] (Table 3).

#### Clinical variables

The clinical problems "urological disorder” and "psychomotor agitation” were significantly associated with the FU condition according to univariate analysis [OR = 2.16 (1.686–2.762) and OR = 7.23 (6.194–8.443), respectively], which was also confirmed by multivariate analysis [OR = 1.85 (1.445–2.376) and OR = 5.67 (4.847–6.636), respectively].

Clinical conditions related to “abdominal pain”, “trauma” and “neurological disorder” reduced the likelihood of FU (Table 4).

#### Care outcomes

The outcomes “not responding to call” and “patient spontaneously leave” were significantly associated with FU status [univariate: OR = 1.94 (1.750–2.145) and OR = 3.94 (3.263–4.746); multivariate: OR = 1.96 (1.759–2.175) and OR = 4.36 (3.596–5.273)]. “Discharge to outpatient facilities” reduces the likelihood of FU and thus reaccessing the PS, findings confirmed by both univariate and multivariate analyses [OR = 0.538 (0.464- 0.622) and OR = 0.684 (0.588–0.796), respectively] (Table 5).

## Discussion

The variables identified as codeterminants of being FUs provide information about geographic and sociodemographic circumstances that contribute significantly to the phenomenon of FUs. In fact, the Municipality VI° is one of the poorest of the city of Rome [13], then the result of the multivariate analysis confirm the role of poor socio-economic resources as a indipendent determinant of being ER’s FUs.

A significant proportion of these admissions are certainly inappropriate and associated with pathologies that can be managed by facilities and health services outside the hospital setting by improving the efficiency of the hospital services provided [17, 18]. As early as the 1980s, the profile of FU patients who accessed the ED because of medical and psychosocial problems was studied. In fact, a Swedish study from 1985 identified the characteristics mainly found in these patients: social isolation, loneliness, repeated contact with social services, disability pension, absenteeism from work due to illness, and alcoholism [19].

Therefore, actions geared toward the establishment, reinforcement and increased supply of territorial/domiciliary services specifically targeting frail, drug-addicted or mentally distressed people [20] could prove to be of utmost benefit in the prevention and control of FUs.

According to the general profile of FU patients, most patients were expected to be elderly, but during this study, a prevalent association was observed with people younger than 65 years [21]. In our FU sample, however, people older than 65 years of age accounted for almost one-third (29.5%) of the population and were hospitalized more frequently.

The authors' hypothesis was to find "older users" for several reasons:

- the progressive aging of the population (in the city of Rome alone the over 65s have increased from 19.7% to 23.5% in the last 20 years) [22];
- in the elderly population there is an increased prevalence of chronic pathologies characterized by more frequent acute exacerbations (62.4% of the over-65s in the Lazio Region, according to the 2021–2022 indicators are suffering from at least 1 chronic disease — > renal failure, chronic bronchitis, emphysema, respiratory failure, bronchial asthma, stroke or cerebral ischemia, diabetes, myocardial infarction, cardiac ischemia or coronary artery disease, other heart diseases, tumors (including leukemia and lymphomas), chronic liver diseases or cirrhosis) [23];
- similar studies conducted at the Tor Vergata Hospital found a prevalence of elderly "frequent users" [24] and demonstrates a change in trend in recent years.

Several published articles agree that one of the factors related to the phenomenon of FUs is limited access to other health care offerings, especially territorial ones.

An Austrian study in 2020 analysing the most frequent causes of ER access, especially in patients who had nonemergency triage codes, highlighted 3 main reasons that led patients to seek ER services: difficulty getting an appointment from their family doctor, being referred to the ER directly by medical office staff, and being too far away from the doctor's office from their home [25].

A systematic review of the literature by Burns et al. showed from these different studies that the two factors that had the greatest impact on frequent ER admissions were lack of education about one's health needs and difficulty accessing medical care [26]. In 2020, J.S. Bittencourt et al. showed that overcrowding in emergency departments is a direct result of an imbalance between the demand and supply of health services offered in the territory and at home.

This is due to poor integration between territorial health, social and welfare networks, and ineffective primary care services, which are objective obstacles to the proper use of hospital services [27].

An experiment conducted in Italy in 2016 hypothesized that extending outpatient clinic hours up to 12 h a day could reduce the inappropriate use of emergency services; the estimated effect, according to Lippi Bruni et al., is on the order of a 10–15% reduction in inappropriate hospitalizations [28].

Our study confirms these observations. In fact, FU patients whose ED accesses resulted in hospitalization tended to reaccess within the year more easily than did those discharged to outpatient facilities, regardless of the clinical and sociodemographic variables considered. The study allows us to hypothesize that discharge to outpatient facilities is a possible marker of all responsive and out-of-hospital alternatives: integrated home care and day care centers in general intermediate care and palliative and concurrent care. Subsequent trials will test this hypothesis.

A systematic analysis of the FUs could identify the main problems of the patient who continually visits the ER. This could be the starting point for public health actions aimed at intercepting and preventing persistent critical issues.

The biggest limitation of this type of study is that the data are extracted from codified information flows, so it is not possible to fully evaluate the patient in his clinical and social complexity.

## Conclusions

In view of what the results of our study also show, it is necessary, in the opinion of the authors, to integrate the new 'intermediate care' structure provided in Ministerial Decree No. 77 of 2022 [29]. It is important to make the system dynamic and capable of moving toward citizens, integrating social support into overall primary and hospital care through the implementation of telemedicine and telemonitoring services.

“The home as the first place of care” [30] is not only a great achievement in mitigating the suffering and discomfort of patients but also an effective antidote to the inappropriate use of hospital facilities.

## Data Availability

The data that support the findings of this study are not openly available due to reasons of sensitivity and are available from the corresponding author upon reasonable request. Data are located in controlled access data storage at Tor Vergata Hospital.

## Abbreviations

CEI: Comitato Etico Indipendente-Independent Ethics Committee
ED: Emergency Department
ER: Emergency Rooms
FUs: Frequent Users
NHS: National Health Service
PTV: Policlinico Tor Vergata
SES: Socioeconomic Status
SD: Standard Deviation
S.I.E.S: Sistema Informativo Emergenze Sanitarie–Health Emergency Information System
PDTA: Diagnostic and Therapeutic Care Pathways
SDO: Scheda di Dimissione Ospedaliera–hospital discharge form
